# Census of Limbless Civilians in Wales

**Published:** 1918-07-06

**Authors:** 


### A Piece of Useful Reconstruction.

### CENSUS OF LIMBLESS CIVILIANS IN WALES.

When the Prince of Wales' Hospital, Cardiff, for Limbless Sailors and Soldiers was opened a year ago the donors stipulated that facilities should be extended to limbless civilians as soon as the demands arising directly from the war permitted. To provide a basis for this prospective work the Lords Lieutenant of the various counties of Wales and Monmouthshire have agreed to help in compiling a full census of limbless for each county.

This census is not yet complete, and a considerable time will probably elapse before the full returns are to hand. The following table shows that the census of eight counties has been completed and a large part of the chief county in the Principality—viz. Glamorganshire.

As the total population of Wales and Monmouthshire at the last census (1911) was 2,420,917 it is evident that there are at least 2,400 limbless civilians. In fact, it is anticipated that the ratio will be found to be quite as high in the four counties and three boroughs from which returns have not yet been received, and that the total figure will be between 2,800 and 3,000.

| | Number of Limbless Civilians | Population |
|---|---|---|
| Anglesey ... ... ... | 19 | 50,928 |
| Brecknockshire ... ... | 67 | 59,287 |
| Cardiganshire ... ... | 44 | 59,879 |
| Carmarthenshire ... ... | 166 | 160,406 |
| Denbighshire ... ... ... | 160 | 144,783 |
| Merionethshire ... ... | 55 | 45,565 |
| Montgomeryshire ... ... | 48 | 53,146 |
| Pembrokeshire ... ... | 89 | 89,956 |
| Total for eight Counties... | 648 | 663,950 |
| Administrative County of Glamorgan (excluding Cardiff, Swansea, Merthyr, and Neath) ... ... | 1,040 | 725,412 |
| Swansea Borough ... ... | 169 | 114,663 |
| Total to April 30 ... | 1,857 | 1,504,025 |

